# Controlling the chaos: Information management in home-infusion central-line–associated bloodstream infection (CLABSI) surveillance

**DOI:** 10.1017/ash.2023.134

**Published:** 2023-04-11

**Authors:** Susan M. Hannum, Opeyemi Oladapo-Shittu, Alejandra B. Salinas, Kimberly Weems, Jill Marsteller, Ayse P. Gurses, Ilya Shpitser, Eili Klein, Sara E. Cosgrove, Sara C. Keller

**Affiliations:** 1 Department of Health, Behavior and Society, Johns Hopkins Bloomberg School of Public Health, Baltimore, Maryland; 2 Department of Medicine, Johns Hopkins University School of Medicine, Baltimore, Maryland; 3 Department of Hospital Epidemiology and Infection Control, Johns Hopkins Hospital, Baltimore, Maryland; 4 Department of Health Policy and Management, Johns Hopkins Bloomberg School of Public Health, Baltimore, Maryland; 5 Armstrong Institute of Patient Safety and Quality, Johns Hopkins University School of Medicine, Baltimore, Maryland; 6 Department of Computer Science, Whiting School of Engineering, Johns Hopkins University, Baltimore, Maryland; 7 Department of Emergency Medicine, Johns Hopkins University School of Medicine, Baltimore, Maryland

## Abstract

**Objectives:**

Access to patient information may affect how home-infusion surveillance staff identify central-line–associated bloodstream infections (CLABSIs). We characterized information hazards in home-infusion CLABSI surveillance and identified possible strategies to mitigate information hazards.

**Design:**

Qualitative study using semistructured interviews.

**Setting and participants:**

The study included 21 clinical staff members involved in CLABSI surveillance at 5 large home-infusion agencies covering 13 states and the District of Columbia. Methods: Interviews were conducted by 1 researcher. Transcripts were coded by 2 researchers; consensus was reached by discussion.

**Results:**

Data revealed the following barriers: information overload, information underload, information scatter, information conflict, and erroneous information. Respondents identified 5 strategies to mitigate information chaos: (1) engage information technology in developing reports; (2) develop streamlined processes for acquiring and sharing data among staff; (3) enable staff access to hospital electronic health records; (4) use a single, validated, home-infusion CLABSI surveillance definition; and (5) develop relationships between home-infusion surveillance staff and inpatient healthcare workers.

**Conclusions:**

Information chaos occurs in home-infusion CLABSI surveillance and may affect the development of accurate CLABSI rates in home-infusion therapy. Implementing strategies to minimize information chaos will enhance intra- and interteam collaborations in addition to improving patient-related outcomes.

Home-infusion therapy is a rapidly growing area of medicine; >3 million patients in the United States receive home and specialty infusion services anually.^1^ These patients and their caregivers commonly maintain central lines in the home and perform the majority of central-line care. They are at risk of central-line–associated bloodstream infections (CLABSIs), just as patients maintaining central lines in other settings would be.^2,3^ Despite this risk of infection, CLABSI surveillance in home-infusion therapy is not systematically performed.^4^

Those who investigate rates of CLABSI in home-infusion therapy, or who perform CLABSI surveillance, face many barriers, including insufficiencies in dedicated or trained staff, lack of benchmarks, and absence of structured reporting processes.^5^ Other major barriers include a lack of clinical information and the difficulty communicating with care teams.^4^ In hospitals, access to data (eg, laboratory results, documented signs and symptoms, and imaging and other reports) is essential, and accessible tools for performing CLABSI surveillance are needed. However, these data may be more difficult to access in home-infusion therapy settings where patients may have been admitted to hospitals with which the home-infusion staff are not affiliated.

This lack of full access to data may contribute to incomplete CLABSI surveillance and incomplete recognition of patient safety risks. Incomplete CLABSI surveillance data can affect how CLABSI surveillance data are interpreted, which has implications for CLABSI benchmarking. In other settings, such as primary care, information hazards described as contributing to patient safety risks have included the following: information underload, necessary information is lacking; information overload, too many data to organize or act on; information scatter, information in multiple places; information conflict, inability to determine which data are correct; and erroneous information.^6^ These information hazards lead to information chaos or information problems that may lead to errors.^6^ Home healthcare professionals may struggle with accessing hospital data and communicating with outpatient providers.^7^ Similar challenges likely apply to home-infusion CLABSI surveillance. Characterizing information chaos in home-infusion therapy is necessary to describe the role of missing data in CLABSI surveillance^8^ and to demonstrate the needed level of support for those doing CLABSI surveillance.^9^

An understanding of contributors to information chaos could affect how CLABSI surveillance data are interpreted, including the possibility of missing data, with implications for CLABSI benchmarking in the home-infusion setting. The objectives of this study were (1) to characterize information hazards in home-infusion CLABSI surveillance and (2) to identify possible strategies to mitigate information hazards.

## Materials and methods

### Research approach

We conducted semistructured interviews with staff involved in CLABSI surveillance (eg, quality staff, administration, infection preventionists, etc) at 5 home-infusion agencies across the United States participating in a CLABSI prevention collaborative, including 2 in the mid-Atlantic, 1 in the Northeast, and 2 in the Midwest, covering portions of 13 states and Washington, DC. All 5 agencies are nonprofit organizations, are affiliated with academic institutions, and serve both community and academic hospitals as well as outpatient providers. Recruitment of study participants was conducted using a combination of purposive and snowball sampling, seeking respondents integral to CLABSI surveillance, and recruiting participants through the collaborative.^10^ We interviewed at least 2 staff members involved in CLABSI surveillance from each of the 5 agencies. Portions of the methods have been previously reported.^5,11^ This study was approved by the Johns Hopkins Institutional Review Board.

### Interview procedures

Using the SEIPS 2.0 framework as a guide, we constructed a semistructured interview guide to explore systems and processes for conducting CLABSI surveillance. We included items querying the following topics: (1) work system of CLABSI surveillance;^12^ (2) tasks and steps for surveillance; (3) barriers and strategies to performing CLABSI surveillance; (4) experience in CLABSI surveillance; and (5) definition of CLABSI used. To ensure consistency in interviewing methods, interviews were conducted by a single team member (S.C.K.). One group interview with 4 respondents was conducted based on an interviewee request and used the same interview guide. After obtaining consent, all interviews were conducted via videoconferencing and lasted 20–60 minutes. Interviews were audio-recorded and were transcribed by a transcriptionist.

### Data analyses

Transcripts were deidentified and uploaded into MAXQDA for data management and analyses (VERBI Software, Berlin, GA). The initial codebook was developed deductively from the interview guide. The deductive codes were applied to the first 3 interview transcripts. In addition, we inductively identified emergent subcodes for each parent code, resulting in a total of 38 subcodes that were applied to understand information management.^6^ This initial coding process was conducted by S.H. and S.C.K.; disagreements in coding were discussed and consensus was reached by mutual agreement.^13^ One coder (S.H.) then coded the remainder of the interview data and both reviewers (S.H. and S.C.K.) discussed emergent findings and modifications to the coding framework. We engaged a process of constant comparison of emergent findings throughout the analytic process, and when no new codes could be identified, we considered thematic saturation to have been achieved.^14^

## Results

**Fig. 1.**
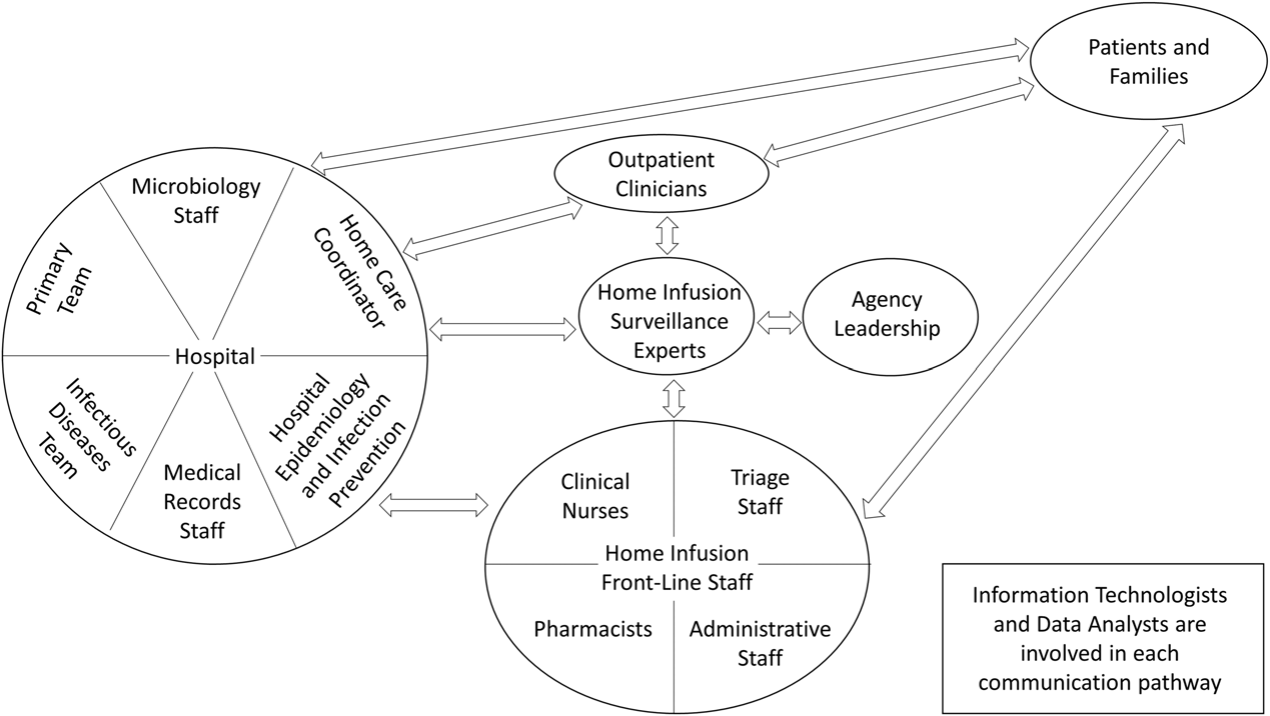
Communication and information exchange pathways in home-infusion CLABSI surveillance.

We interviewed 21 individuals between November 2020 and April 2021. The respondents were primarily nurses (N = 18, 86%) (Table 1). We describe barriers impeding the flow of information necessary for effective CLABSI surveillance (Table 2), and we discuss communication pathways required for successful CLABSI surveillance in home infusion (Fig. 1). Finally, we present recommendations for improving communication and information access in home-infusion CLABSI surveillance (Table 3).

**Table 1.** Participant Descriptions

Characteristic	Total (N = 21), No. (%)
**Home infusion agency**	
Agency 1	6 (29)
Agency 2	5 (24)
Agency 3	6 (29)
Agency 4	2 (10)
Agency 5	2 (10)
**Sex**	
Male	4 (19)
Female	17 (81)
**Race**	
White	19 (90)
Black	1 (5)
Asian American	1 (5)
Ethnicity non-Hispanic^a^	18 (86)
**Role**	
Nurse	18 (86)
Pharmacist	3 (14)

a 3 did not report.

**Table 2.** Information Barriers and Suggested Strategies for Performing Home-Infusion CLABSI Surveillance

Type of Information Barrier	Example(s) of Information Barrier(s)	Strategy(ies) to Mitigate Information Barrier(s)
Information underload	Unknown what patients to follow No process in place to get information in a standardized way Delay in information being provided Hospital staff do not speak to home-infusion agencies due to misunderstanding of HIPAA Incomplete information being entered into home-infusion EHR Home-infusion clinical staff not aware of CLABSIs	Electronic reports Develop process for information Train patient as communicator Train triage staff as communicator Timely reporting of complications Gain access to EHRs of hospitals to which patients are admitted
Information overload	Too much information to read through when finding data Screening reports are overly broad and include data on patients who do not meet CLABSI criteria.	Electronic reports Clarifying which information should be placed in which locations in the EHR
Information scatter	Multiple EHRs Variable access to EHRs Information recorded in multiple formats Multiple methods of communication	Have 1 dashboard to record all information Develop reports with relevant information from the EHR Use of EHR to create shared communication Using shared videoconferencing and document sharing technology
Information conflict	Recorded as having CLABSI in chart by physician without meeting surveillance definition Different definitions of CLABSIs	One common surveillance definition Understanding the surveillance definition
Erroneous information	Recorded as culture not complete but culture in another location is complete.	Collaboration with staff at hospitals Requesting final data

Note. EHR, electronic health record; CLABSI, central-line–associated bloodstream infection.

**Table 3.** Key Recommendations for Addressing Information Chaos in Home-Infusion CLABSI Surveillance


Key Point	Recommendations for Addressing Information Chaos in Home-Infusion CLABSI Surveillance
1	Engage information technology and data analysts in developing accurate reports of patients with central venous catheters and duration of central venous catheters for denominator calculations, and of patients with potential CLABSIs.
2	Develop clear, streamlined processes for acquiring and sharing data among members of the home-infusion therapy agency and the home-infusion surveillance staff with shared dashboards, files, and reporting mechanisms.
3	Enable home-infusion surveillance staff access to EHRs of hospitals to which their patients are commonly admitted.
4	Use a single, validated home-infusion CLABSI surveillance definition.
5	Develop relationships between home-infusion surveillance staff and those with knowledge of the inpatient course of patients to enable surveillance staff access to additional data.

Note. EHR, electronic health record.

### Information underload

Information underload (ie, when necessary information is lacking) was common. Which patients surveillance staff needed to monitor (ie, which patients had central lines) was unclear, and it was difficult to access data required for CLABSI adjudication. Acquiring these data took time, and there was uncertainty about their quality. Information underload also resulted from difficulties communicating with hospital staff, who had details useful in determining whether a patient had been admitted with a CLABSI. Some hospital staff members did not speak to home-infusion surveillance staff because they did not understand the role of home-infusion surveillance staff in patient care.

Information underload also occurred at an agency-wide level. Although home-infusion surveillance staff typically reported CLABSIs to leadership, these data often were not shared with frontline clinicians. As a result, many frontline clinicians did not know when home-infusion CLABSIs occurred.

To address information underload, home-infusion CLABSI surveillance staff developed processes to obtain information, such as communicating with individuals at hospitals. Some used workarounds, such as using a patient or family member to get information. Others received information from triage staff or frontline clinicians making reports if a patient had a complication. Home-infusion surveillance staff needed better reports to know which patients to follow for a potential CLABSI. An infusion pharmacist leader at agency 1 explained:

“[The infusion staff] also gets reports from [the hospital system] for patients that are readmitted or have diagnosis of CLABSI and she does a reconciliation between those reports and our census … Not the most ideal workflow or process or the easiest one to manage at the moment, so we’re constantly looking for better ways to pull that information and gather that data more automatically.”

A key takeaway was to engage information technology and data analysts in developing accurate reports of duration of central lines for denominator calculations as well as accurate reports of patients with potential CLABSIs.

### Information overload

Information overload (when there are too many data to organize or act on) also occurred in home-infusion CLABSI surveillance activities. At times, too much information was available, including information that was not relevant. Reports used by surveillance staff to investigate patients for possible CLABSIs were sometimes overly broad (including all patients who were hospitalized, for example) and included patients who did not have bacteremia. Many informants said that there was too much information to process when finding data, partially due to a lack of a process for tracking data and no summarization of narrative data. A pharmacist from agency 5 explained: “All of our, quote, tracking in the pharmacy system right now is just progress notes and our narrative on the patient chart.” A major outcome of such information overload was significant wasted time, in a setting where many participants felt that they did not have sufficient allotted time to complete the main tasks of their job. Some even described relevant data being overlooked.

To prevent information overload, respondents described developing clear, streamlined processes for acquiring and sharing data among members of the home-infusion agency and the home-infusion surveillance staff, incorporating shared dashboards, files, and reporting mechanisms, and evaluating critically what data are most relevant. As one nurse at agency 3 described,

“[Our EHR has] pop-ups … and… they’ve been very cluttered and information has been in there that’s not relevant to most people and, anyways, it’s caused opportunities for things to get missed, so we’[re] work[ing] on what actually should be in there.”

### Information scatter

Information scatter (information in multiple places) was described as a barrier to effective communication throughout all phases of CLABSI surveillance. Home-infusion surveillance staff reported having to access information from e-mails, multiple EHRs, and telephone calls. Some staff members did not have access to EHRs of all hospitals to which their patients were admitted and had to depend on others to get information. As an IP at agency 1 stated, data in home-infusion was less standardized than hospital data: “I mean, you know how hard it is to get those reports from a standardized group of nurses that are caring for all of your patients in the hospital in one place.”

To address information scatter, an infusion pharmacist at agency 5 suggested having 1 data collection template to collect the data: “I think [a data collection tool] sounds amazing, to have 1 place to maybe just make a couple clicks here and there and be able to collect accurate data and the most complete data.” Respondents also recommended using the EHR for communication and to create reports of patients with potential complications. Interviewees suggested reducing information scatter through technologies such as videoconferencing, team-based technology, using delivery tracking tickets, shared dashboards, files, and reporting mechanisms. Another suggestion was to enable home-infusion surveillance staff access to EHRs of hospitals to which patients are commonly admitted.

### Information conflict

Information conflict (ie, the inability to determine which data are correct) was another noted barrier in home-infusion surveillance. A major example of information conflict was when a clinician recorded a CLABSI in a medical record without it necessarily meeting CLABSI surveillance criteria. This commonly occurred, as clinical definitions may differ from surveillance definitions. However, many home-infusion surveillance staff took clinicians’ clinical judgement of a CLABSI for surveillance purposes. Many explained that the lack of a standardized CLABSI definition worsened information conflict, as a surveillance staff member from agency 4 explained:

“We have tried different tools that are out there to make sure that when you do CLABSIs, it has to be apples to apples, and it is really challenging … so honestly, we look at the notes by infectious disease, and infectious disease will rule out and do their thing and if they say the line is the source of the infection then we call it a CLABSI.”

Participants described the utility of the potential for a single, validated home-infusion CLABSI surveillance definition. A quality manager at agency 1 explained:

“Because we don’t have a solid definition we cannot judge whether the CLABSI is confirmed or not…. [if] that was the appropriate CLABSI definition … or not …”

### Erroneous information

Finally, erroneous information (when information is incorrect) occurred in home-infusion CLABSI surveillance, leading to incorrect CLABSI reports. For example, if records were transmitted prior to cultures being complete, the surveillance staff may believe erroneously that a patient had negative blood cultures, as a pharmacist at agency 5 stated: “[Sometimes] we don’t get the final culture results, so we’re not able to put it into the actual … EMR.” Developing relationships between home-infusion surveillance staff and those with knowledge of the inpatient course of patients was one way to support surveillance staff in accessing additional patient data.

## Discussion

Information chaos may contribute to decreased situation awareness about the possibility of CLABSIs.^6,15^ Because agencies may be unaware of CLABSI rates, they may not act consistently to prevent CLABSIs. In addition, the increase in mental workload may lead to mistakes or burnout. Information chaos may impede accurate surveillance and requires a system to unify data collection. Mitigating this information chaos is vital to the performance of surveillance staff, the performance of the agency, and the agency’s ability to enhance patient safety by being able to address CLABSIs. We developed 5 key recommendations that could increase the accuracy and simplicity of CLABSI surveillance (Table 3).

First, we showed that home-infusion CLABSI surveillance should engage both information technology and data analyst professionals to identify which patients need to be followed for CLABSIs and which may already have a CLABSI. A study of 10 home-infusion agencies collaborating to report CLABSIs noted that several agencies did not have a pre-existing system to track complications, and few used automated reports.^16^ Although electronic data alone have not yet proven adequate for CLABSI surveillance,^17^ developing reports of eligible patients is an essential part of CLABSI surveillance. Hospital EHRs contain modules to aid in identifying which patients have central venous catheters, to calculate denominator data, and to determine who has positive blood cultures.^18^ Information technology (IT) and data-analyst support from hospitals and home-infusion agencies could also aid in home-infusion CLABSI surveillance. For example, IT reports used to support hospital-based infection prevention could be modified to aid in home-infusion CLABSI surveillance. However, EHRs may not always be designed to support the home health provider workflow.^7^ Therefore, home-infusion CLABSI surveillance reports and tools should be developed or modified to fit the home-care workflow.^19^ Health system or hospital-based infection prevention departments could aid affiliated home-infusion agencies in developing these reports and tools to increase efficiencies.

Second, we identified that home-infusion agencies would benefit from shared communication structures and streamlined processes for acquiring and sharing data among team members. This would aid the home-infusion surveillance staff with getting information on patients with possible CLABSIs as well as providing information about CLABSIs to home-infusion staff. Although the EHR could be considered a central method to mitigate information chaos,^6,7^ understanding how to design the EHR to facilitate this data exchange is essential.^20^ Team-based electronic dashboards and communication platforms may be required outside the EHR to allow team members to easily exchange IP concerns.

Third, home-infusion surveillance staff should be given access to the EHR of hospitals to which patients are commonly admitted. EHR data are essential to understanding the hospital course of a patient diagnosed with a complication, and these data are also important in home-health information management.^7^ However, home-infusion patients can be admitted to hospitals with different EHR systems. Some EHR platforms and even states allow data sharing through health information exchanges.^21,22^ Home-infusion agencies should be provided access to these health information exchanges.

Fourth, we identified the importance of a validated, benchmarked home-infusion CLABSI surveillance definition. A 2008 home healthcare–associated bloodstream infection definition^23^ relied on National Healthcare Safety Network (NHSN) acute-care CLABSI surveillance criteria in use at the time,^24^ but it has not been widely adopted.^25^ A proposed home-infusion CLABSI surveillance definition still requires validation prior to larger use.^26^ A validated, widely accepted, home-infusion CLABSI surveillance definition will enable communication among home-infusion surveillance staff.

Fifth, we learned that home-infusion CLABSI surveillance staff could benefit from building relationships with individuals at hospitals, such as healthcare epidemiologists or infection preventionists who may be able to provide information or context. Others have noted the need to bring hospital-based and home-based staff together to understand information needs and to redesign information-sharing tools between hospital-based and home-based staff.^7,27,28^ We suggest that this information redesign should also occur in home infusion therapy.

Our findings have implications for calculations of CLABSIs rates in home infusion therapy. Each of the information barriers identified could lead to missing data, which could lead to either an underestimation or overestimation of the CLABSI rate. This misestimation may affect perceptions of CLABSIs in home infusion therapy. In a national survey, some home-infusion professionals stated that they did not see CLABSIs as a problem in their setting.^4^ Our study is one of the first analyses of information management in HAI surveillance. Others could use these models to investigate information flows in HAI surveillance in other settings.

Our study had several limitations. The participants were all members of 5 nonprofit home-infusion agencies in the mid-Atlantic, Midwest, and Northeastern United States, each of which is affiliated with or owned by an academic health system, which increases the likelihood of selection bias and limits generalizability. Specific tasks required for CLABSI surveillance may be different in for-profit agencies, agencies in other parts of the country, or in those not affiliated with an academic health system. However, participants worked in home-infusion agencies in 13 different states. Because we focused primarily on CLABSI surveillance, we did not focus interviews on those not directly involved in surveillance, so we interviewed fewer agency leaders or frontline clinicians. In addition, because these agencies have agreed to participate in a collaborative focused on CLABSI surveillance and prevention, participants may have been more experienced with CLABSI surveillance than other agencies. It is possible that information management practices may be different at nonparticipating agencies.

We suggest a focus on mitigating inadequate communication structures by increasing access to information technology or data analyst specialists, improving intra-agency communication structures, increasing access to hospital EHRs. Also, a validated home-infusion CLABSI surveillance definition and closer relationships with home-infusion surveillance staff and inpatient personnel are needed. We suggest that the possibility of missing data be considered in analyzing the burden of home-infusion CLABSIs. Finally, we suggest a greater focus on home-infusion CLABSI surveillance to understand the rates of CLABSI in home infusion therapy. Health systems should work to support the information flows required for appropriate home-infusion CLABSI surveillance to ensure that CLABSI prevention activities can be initiated.
